# Validation of online psychometric instruments for common mental health disorders: a systematic review

**DOI:** 10.1186/s12888-016-0735-7

**Published:** 2016-02-25

**Authors:** Wouter van Ballegooijen, Heleen Riper, Pim Cuijpers, Patricia van Oppen, Johannes H. Smit

**Affiliations:** Department of Psychiatry, VU Medical Centre / GGZ inGeest, Amsterdam, Netherlands; Department of Clinical Psychology, Vrije Universiteit Amsterdam, Amsterdam, Netherlands; EMGO Institute for Health and Care Research, VU Medical Centre, Amsterdam, Netherlands

**Keywords:** Internet, Depression, Anxiety, Measurement, Psychometrics, Systematic review

## Abstract

**Background:**

Online questionnaires for measuring common mental health disorders such as depression and anxiety disorders are increasingly used. The psychometrics of several pen-and-paper questionnaires have been re-examined for online use and new online instruments have been developed and tested for validity as well. This study aims to review and synthesise the literature on this subject and provide a framework for future research.

**Methods:**

We searched Medline and PsycINFO for psychometric studies on online instruments for common mental health disorders and extracted the psychometric data. Studies were coded and assessed for quality by independent raters.

**Results:**

We included 56 studies on 62 online instruments. For common instruments such as the CES-D, MADRS-S and HADS there is mounting evidence for adequate psychometric properties. Further results are scattered over different instruments and different psychometric characteristics. Few studies included patient populations.

**Conclusions:**

We found at least one online measure for each of the included mental health disorders and symptoms. A small number of online questionnaires have been studied thoroughly. This study provides an overview of online instruments to refer to when choosing an instrument for assessing common mental health disorders online, and can structure future psychometric research.

**Electronic supplementary material:**

The online version of this article (doi:10.1186/s12888-016-0735-7) contains supplementary material, which is available to authorized users.

## Background

Assessment of common mental health disorders, which include depression and anxiety disorders [1], is increasingly conducted online, usually employing self-report questionnaires. Current online instruments are often paper questionnaires that have been adapted for online use [2]. Paper and online versions of the same instrument correlate strongly, but mean scores and psychometrics may differ [2] and, therefore, equivalence cannot be assumed.

Several studies have re-examined the psychometrics of paper questionnaires for use online, e.g. for measuring social phobia [3], panic and agoraphobia [4] and depression [5]. Besides the established paper instruments that are used online, new instruments are being developed and investigated for validity specifically for use online. These instruments can have technological advantages, such as the use of audio and video [6, 7], or automatically skipping irrelevant items based on previous answers [8].

To date, the psychometrics of both the digitalised paper questionnaires and newly developed online instruments have not been systematically studied. An overview and synthesis of the literature would provide a framework for future research and development, and would guide researchers, clinicians and other professionals when choosing an instrument suitable for a specific purpose. The current study aims to systematically review and synthesise the scientific literature on the psychometrics of internet-based instruments that measure common mental health disorders and related symptoms. We aim to provide an overview of the psychometric characteristics of these instruments, the evidence for these characteristics, and an indication of how these findings can be generalised to various populations.

## Methods

This systematic review was conducted in accordance with the PRISMA Statement [9]. See Additional file 1. The extraction of psychometric data was based on the COnsensus-based Standards for the selection of health status Measurement Instruments (COSMIN) Checklist. [10]

### Study selection

We conducted a comprehensive literature search in PubMed and PsycInfo, which is updated up to January 1st 2014. For the PubMed search we applied a previously developed search string for psychometric studies [11] and additional key words to focus on online assessment and common mental health disorders (Additional file 2). The PsycInfo search was a translation of the PubMed search, with additional keywords unique to PsycInfo and the omission of generic terms such as ‘methods’ and ‘instrumentation’, to increase the specificity of the search (Additional file 2).

### Study inclusion

**Fig. 1:**
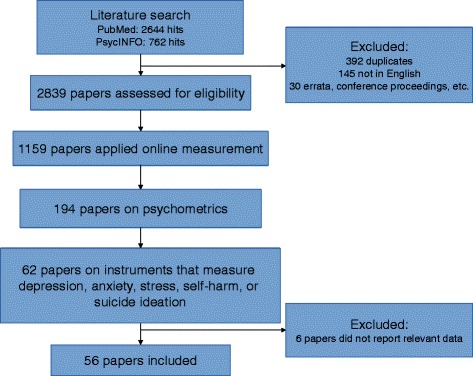
Flow chart of included studies

After excluding studies that were not written in English, studies were included in three a priori defined steps, as depicted in the flow chart (Fig. 1). The first inclusion step was to select all studies that applied online self-report assessments, i.e. data were collected using internet-connected devices that individuals used to fill in questions about themselves. We excluded assessments through stand-alone devices (e.g. in a clinic), or other self-report measurement within a clinic, in order to retain comparability between results. We also excluded studies on assessments through unique devices specifically developed for the study, face-to-face interviews conducted by videoconference, and interactive voice response measures by telephone. As second inclusion step, we included only those studies that aimed at assessing psychometrics and that provided data of at least one psychometric variable. The third and final inclusion step included studies that described instruments for assessing symptoms of common mental health disorders [1]. These disorders include ICD-10 [12] and DSM-5 [13] unipolar depressive disorders, social phobia, panic disorder with or without agoraphobia (PD/A), agoraphobia without panic, specific phobia, generalised anxiety disorder (GAD), post-traumatic stress disorder (PTSD) and obsessive-compulsive disorder (OCD). We also included instruments that assessed specific symptoms of these disorders or general distress that can accompany these disorders, i.e. psychological stress (only when unrelated to physical disorders), worrying, suicidal ideation and self-harm.

### Data extraction

First, we coded the data that are relevant for generalising a study’s findings, which are the sample size, characteristics of the participants (age, gender, disease characteristics), population (e.g. patients or general population), recruitment method, country in which the study was conducted, language of the measurement instrument, any subgroups the results were reported for, and amount of missing data. Next, we extracted the psychometric data provided in the study. The following variables were entered into the tables (Tables 1, 2, 3 and 4; Additional file 3): internal consistency (usually expressed as Cronbach’s alpha); test-retest reliability (usually kappa); measurement error; factor structure, including type of analysis (exploratory or confirmatory factor analysis, or principal component analysis) and model fit or variance explained; equivalence of paper and online versions of the instrument (usually a correlation); difference in mean scores between online and paper versions; convergent validity, i.e. the relation with an instrument that measures the same construct (usually a correlation); criterion validity in terms of sensitivity, specificity (for the optimal cut-off point), Area Under the Receiver Operating Characteristic Curve (AUC), and other criterion-related outcomes (e.g. kappa); and responsiveness, i.e. the degree to which the instrument can measure change. These variables were extracted for each instrument reported in the study. When an instrument was investigated in multiple samples, e.g. when two studies on one instrument were described in one paper, we listed the sample characteristics and outcomes for each sample separately.

**Table 1:** Transdiagnostic online self-report instruments and the number of studies that report psychometric characteristics (between parentheses)

Instrument	Purpose	N studies	Population / setting	Countries in which the studies were conducted	Internal consistency (alpha)	Test-retest reliability	Factor structure	Mean score difference with paper version	Convergent validity	Criterion validity (AUC)
Anxiety										
BAI	Symptom severity	3	G1, P3	SE	.88–.89 (2)		4 factors (1)	None, lower (2)		
Depression and anxiety										
CIDI-SF	Diagnosis & screening	1	G1	SE						(1)
DASS	Symptom severity	1	G5	US	.93–.95 (1)	(1)			(1)	
HADS	Screening & symptom severity	5	G1, G5, P5, P7	NZ, SE, UK	.76–.88 (5)	(1)	3 factors (2)	None (2)	(1)	
SQ-48	Screening & symptom severity	1	G1, P1	NL	.84–.93 (1)		9 factors (1)		(1)	.75–.91 (1)
WB-DAT	Screening	1	P8	CA						(1)
WSQ	Screening	1	G1	NL						.65–.81 (1)
Depression and anxiety (postpartum)										
PDM	Screening	1	G1	US	.84–.88 (1)		2 factors (1)		(1)	

G1: General population; G2: Adult females; G3: Adult males; G4: General teenage population; G5: Student population; G6: General young adult population; G7: Veteran population; P1: Patient population; P2: Adult psychiatric outpatients; P3: Adult social phobia patients; P4: Adult GP patients; P5: Hearing impaired clinical population; P6: Deaf population; P7: Adults with chronic fatigue syndrome; P8: Participants in studies of the Centre for Addiction and Mental Health; AU: Australia; CA: Canada; DK: Denmark; ES: Spain; NL: Netherlands; NO: Norway; NZ: New Zealand; SE: Sweden; TW: Taiwan; UK: United Kingdom; US: United States; AUC: Area Under the Receiver Operating Characteristic Curve

**Table 2:** Online self-report instruments for depression and the number of studies that report psychometric characteristics (between parentheses)

Instrument	Purpose	N studies	Population / setting	Countries in which the studies were conducted	Internal consistency (alpha)	Test-retest reliability	Factor structure	Mean score difference with paper version	Convergent validity	Criterion validity (AUC)
Depression										
BDI	Symptom severity	2	G1	SE	.88 (1)		3 factors (1)	Higher (1)		
BDI-II	Symptom severity	2	G1	SE	.87–.95 (2)			None, lower (2)		
CES-D	Screening & symptom severity	6	G1, G2, G4, G5	NL, TW, US	.89–.93 (5)		2–4 factors (2)	None (2)	(2)	.84–.90 (2)
CES-D (7-item)	Symptom severity	1	G5	ES	.82 (1)		1 factors (1)	None (1)	(1)	
CES-D (10-item)	Symptom severity	1	G1	US	.86 (1)					
CUDOS	Screening & symptom severity	1	P2	US	.93 (1)			None (1)	(1)	
EDS	Screening	1	G1	NL	.87 (1)				(1)	
HSCL-10	Screening	1	G4	DK + NO	.87 (1)					.79 (1)
ISP-D	Screening	2	G1	TW		(1)				(1)
K-10	Screening & symptom severity	1	G1	NL	.90 (1)				(1)	.81 (1)
K&D mood scale	Symptom severity	1	G5	US	.75–.79 (1)	(1)			(1)	
MADRS-S	Symptom severity	6	G1, P3	SE	.73–.90 (5)		3 factors (1)	None (4)		
MDI	Screening & symptoms severity	1	G4	NL	.82 (1)				(1)	.89 (1)
MDRS-22	Screening & symptom severity	1	G3	AU			6 factors (1)		(1)	
Moodscope	Symptom severity	1	P4	UK					(1)	
PDI MDD	Diagnosis & screening	1	P1	US					(1)	(1)
PHQ-9 BSL	Symptom severity	1	P6	UK	.81 (1)		2 factors (1)		(1)	
Single item depr. scale	Screening & symptom severity	1	G1	NL					(1)	.71 (1)
USDI	Symptom severity	1	G5	AU	.95 (1)		3 factors (1)		(1)	
ZDS	Symptom severity	1	G5	UK	.89 (1)				(1)	
Postpartum depression										
EPDS	Screening, symptom severity	1	G2	unclear	.90 (1)		3 factors (1)		(1)	
PDSS	Screening & symptom severity	1	G2	US	.97 (1)				(1)	

G1: General population; G2: Adult females; G3: Adult males; G4: General teenage population; G5: Student population; G6: General young adult population; G7: Veteran population; P1: Patient population; P2: Adult psychiatric outpatients; P3: Adult social phobia patients; P4: Adult GP patients; P5: Hearing impaired clinical population; P6: Deaf population; P7: Adults with chronic fatigue syndrome; P8: Participants in studies of the Centre for Addiction and Mental Health; AU: Australia; CA: Canada; DK: Denmark; ES: Spain; NL: Netherlands; NO: Norway; NZ: New Zealand; SE: Sweden; TW: Taiwan; UK: United Kingdom; US: United States; AUC: Area Under the Receiver Operating Characteristic Curve

**Table 3:** Online self-report instruments for GAD, panic disorder and agoraphobia, social phobia, specific phobia, OCD and PTSD, and the number of studies that report psychometric characteristics (between parentheses)

Instrument	Purpose	N studies	Population/setting	Countries in which the studies were conducted	Internal consistency (alpha)	Test-retest reliability	Factor structure	Mean score difference with paper version	Convergent validity	Criterion validity (AUC)
GAD										
GAD-1	Screening	1	G1	NL						.78 (1)
GAD-2	Screening	1	G1	NL						.76 (1)
GAD-7	Screening & symptom severity	1	G1	NL	.86 (1)		1 factor (1)		(1)	.77 (1)
GAD-7 BSL	Symptom severity	1	P6	UK	.88 (1)		1 factor (1)		(1)	
PDI GAD	Diagnosis & screening	1	P1	US					(1)	(1)
Panic disorder and agoraphobia										
ACQ	Symptom severity	2	G1	AU, SE	.81–.84 (2)			None, lower (2)		
BSQ	Symptom severity	2	G1	AU, SE	.84–.86 (2)			None, lower (2)		
CIDI-Panic subscale	Diagnosis & screening	1	G1	SE						(1)
MI	Symptom severity	2	G1	AU, SE	.94–.97 (2)			None, higher (2)		
PDSS-SR item 4	Screening	1	G1	NL						.68 (1)
PDSS-SR item 5	Screening	1	G1	NL						.61 (1)
VS-CMD-agoraphobia	Screening	1	G1	NL						.73 (1)
Social phobia										
LSAS-SR	Symptom severity	2	G1, P3	SE	.93–.94 (2)			None (1)		
SIAS	Symptom severity	3	G5, P3	SE, US	.86–.93 (3)		1 factor (1)	None (2)	(2)	
SPIN	Symptom severity	1	G1	TW	.94 (1)	(1)	3 factors (1)			.87 (1)
SPS	Symptom severity	2	G5, P3	SE, US	.89–.93 (2)		1 factor (1)	None (2)	(1)	
Specific phobia (aviophobia)										
FAS	Screening & symptom severity	1	G1 + G5	US	.94–.99 (1)					.99 (1)
OCD										
C-FOCI	Screening	1	G4	US	.73 (1)					
OBQ-44	Symptom severity	1	G5	US	.97 (1)			None (1)	(1)	
OCI	Symptom severity	1	G5	US	.94 (1)			None (1)	(1)	
PI	Symptom severity	1	G1	US (mainly)			4 factors (1)			
PTSD										
NSES	Diagnosis & symptom severity	1	G1, G7	US			4 factors (1)			
PCL-C	Symptom severity	1	G5	US	.91 (1)			None (1)		
PSS	Screening & symptom severity	1	G5	US	.92–.94 (1)		4 factors (1)		(1)	
TSS	Symptom severity	1	G5	US	.95–.96 (1)			None (1)		
PTSD (peripartum)										
PPQ (modified)	Screening & symptom severity	1	G2	US	.90 (1)		3 factors (1)		(1)	(1)

G1: General population; G2: Adult females; G3: Adult males; G4: General teenage population; G5: Student population; G6: General young adult population; G7: Veteran population; P1: Patient population; P2: Adult psychiatric outpatients; P3: Adult social phobia patients; P4: Adult GP patients; P5: Hearing impaired clinical population; P6: Deaf population; P7: Adults with chronic fatigue syndrome; P8: Participants in studies of the Centre for Addiction and Mental Health; AU: Australia; CA: Canada; DK: Denmark; ES: Spain; NL: Netherlands; NO: Norway; NZ: New Zealand; SE: Sweden; TW: Taiwan; UK: United Kingdom; US: United States; AUC: Area Under the Receiver Operating Characteristic Curve

**Table 4:** Online self-report instruments for stress, worrying, suicidal ideation and self-harm, and the number of studies that report psychometric characteristics (between parentheses)

Instrument	Purpose	N studies	Population/setting	Countries in which the studies were conducted	Internal consistency (alpha)	Test-retest reliability	Factor structure	Mean score difference with paper version	Convergent validity	Criterion validity (AUC)
Stress										
PSS	Symptom severity	1	G5	ES	.72 (1)		1 factor (1)	None/lower (1)	(1)	
Worry										
PSWQ	Symptom severity	2	G1, G5	NL, US	.73–.88 (2)	(1)	1 factor (1)		(1)	
Worry (postpartum)										
PWS-R	Symptom severity	1	G2	US	.64–.88 (1)		4 factors (1)		(1)	
Self-harm										
ISAS	Symptom severity	1	G6	US + UK + CA + AU	.77–.87 (1)		2 factors (1)			(1)
Suicidal ideation										
BDI-II item 9	Screening	1	G1	SE				Lower (1)		
MADRS-S item 9	Screening	1	G1	SE				None (1)		

G1: General population; G2: Adult females; G3: Adult males; G4: General teenage population; G5: Student population; G6: General young adult population; G7: Veteran population; P1: Patient population; P2: Adult psychiatric outpatients; P3: Adult social phobia patients; P4: Adult GP patients; P5: Hearing impaired clinical population; P6: Deaf population; P7: Adults with chronic fatigue syndrome; P8: Participants in studies of the Centre for Addiction and Mental Health; AU: Australia; CA: Canada; DK: Denmark; ES: Spain; NL: Netherlands; NO: Norway; NZ: New Zealand; SE: Sweden; TW: Taiwan; UK: United Kingdom; US: United States; AUC: Area Under the Receiver Operating Characteristic Curve

Criterion validity requires a criterion such as a diagnosis that can be objectively measured, but there is no exact method to ascertain any of the included disorders. Nevertheless, some psychometric studies do aim to assess criterion validity, and the criterion is established by an interview conducted face-to-face or by telephone by a clinician or a trained interviewer. We report these data, because it is not within the scope of the present review to discuss the validity of the used criteria.

### Quality assessment

Quality assessment was conducted in two ways. First, we coded variables that affect the generalisability and risk of bias of the findings, which are sample size, sample characteristics, recruitment method and amount of missing data. Second, we used the COSMIN Checklist with a 4-point scale [10, 14–17]. This checklist contains quality criteria for the psychometric variables described above. For each variable, a quality score is obtained by taking the lowest rating of any item in that list of criteria [10].

### Double coding

The inclusion process was conducted by two independent raters (WvB and a research assistant). Differences between raters were solved by discussion and by conferring with the co-authors. Three of the authors of this study (WvB, JHS, PvO) and three other raters (research assistants) participated in the data extraction and quality assessment of the included studies. We double coded all extracted data, including the four variables that may affect generalisability and risk of bias. The COSMIN quality ratings were mostly single coded, where 18 % of the included studies were double coded.

### Data synthesis

All extracted data (Additional file 3) were sorted on disorder or symptom and on instrument name, thereby creating a table of instruments for each disorder (Tables 1, 2, 3 and 4). When a study investigated multiple instruments for multiple disorders (e.g. one instrument that measures depression and one that measures anxiety), we reported the instruments in the table for the appropriate disorder. We created a separate table for instruments that measured multiple disorders or general symptoms. It was not possible to synthesise the data in a quantitative analysis, such as a meta-analysis, because the included studies investigated a variety of instruments, applying various methods to obtain psychometric data and reporting various outcome measures.

## Results

### Study selection

The PubMed search yielded 2644 results and the PsycINFO search added 370 unique studies (Fig. 1). After excluding studies that were not in English and search results that were irrelevant studies, we assessed 2839 studies for eligibility (Fig. 1). Step one of the inclusion, i.e. assessment was conducted using online self-report instruments, left 1159 studies. Of these, 194 investigated and reported psychometric data (step 2). Next, we included 62 studies that investigated instruments for assessing common mental health disorders (step 3). Finally, we excluded 6 studies that did not report psychometric data that were relevant for our overview and synthesis, so we included 56 studies in our review. See Fig. 1 for a flow chart.

### Study characteristics

The details of the 56 included studies and their results are presented in Additional file 3. Combined, these studies described psychometric data for 62 different instruments. These studies and instruments are presented in Additional file 3. The data are summarised in Tables 1, 2, 3 and 4. The samples of most studies (48 of 56) contained a larger percentage of women (range 0 % to 100 %; Additional file 3). Seven studies included a sample with an average age below 20. Most studies recruited their samples from the general population using advertisements or links on websites (i.e. self-referral). Also common were studies among university students. Patient populations were less common, as 14 of the 62 instruments were investigated among patient populations. See Tables 1, 2, 3 and 4 and Additional file 3. All 56 of the included studies investigated internet-based instruments that were completed on a desktop, laptop or tablet computer, while none of the studies reported that their instruments were completed on cellular phones or smartphones.

### Outcomes

We found instruments for all of the included mental health disorders. An average of 2.5 psychometric characteristics were reported for each instrument. None of the studies reported measurement error or responsiveness of instruments. We left the empty columns of these two outcomes in Additional file 3, but omitted them in Tables 1, 2, 3 and 4. Of the 62 investigated instruments, 29 assessed depressive symptoms. Of these, the CES-D and the Montgomery–Åsberg Depression Rating Scale Self Report (MADRS-S) were most frequently studied (6 studies each). Least studied were instruments for measuring suicidal ideation (1 study on 2 single items), self-harm (1 study) and stress (1 study).

#### Transdiagnostic online instruments

Seven instruments assessed both depressive and anxiety symptoms, or anxiety symptoms that apply to several disorders, such as the Beck Anxiety Inventory (BAI). These can be roughly divided in short instruments that screen for disorders, e.g. the Web Screening Questionnaire (WSQ) [18] and the Web-Based Depression and Anxiety Test (WB-DAT) [8], and scales that assess symptom severity, e.g. the Hospital Anxiety and Depression Scale (HADS) [19] and the Depression Anxiety Stress Scales (DASS) [20]. The short screening questionnaires had poor to adequate criterion validity for screening individual disorders [8, 18, 21]. Of the symptom severity scales, the HADS was investigated in 5 studies [19, 22–25]. These 5 studies showed a fair to good internal consistency. The online HADS is the only instrument we found that was investigated among several patient populations [19, 23, 24]. Although the factor structure may be different from how the measure was designed [19, 23], there is mounting evidence that support adequate validity of the online HADS.

#### Online assessment of depression

Our review includes 29 instruments that measure depressive symptoms. These consist of 22 instruments that measure depression alone and 7 transdiagnostic instruments. The 22 studies on instruments for depression generally reported recruiting their samples from the general population. Five studies investigated instruments for depression among patient populations [3, 6, 26–28], each investigating a different instrument.

The full version of the CES-D has been evaluated in 6 studies [5, 29–33], and 5 characteristics were each reported by at least 2 studies (Table 2). Moreover, all 6 studies recruited their samples among non-patients, so the results can be considered complementary. The internal consistency was investigated in 5 of these studies, reporting a Cronbach’s alpha of .89 to .93. Factor analysis showed that the CES-D consists of 2, 3 or 4 factors [32, 33]. The 2-factor solution was among an English speaking population, the 3-factor solution among a Spanish speaking and the 4-factor solution among a Chinese speaking population [32, 33]. Adequate psychometric characteristics were found for the CES-D regarding equivalence of mean scores with the paper version [31, 33], convergent validity [5, 30] and criterion validity [5, 30]. One study [33] conducted a full measurement invariance analysis using confirmatory factor analysis, comparing paper and online formats, and found only a negligible difference in the latent mean score of one factor. Overall, it can be concluded that the online CES-D has good psychometric characteristics among non-patient populations, and that a start has been made to investigate its intercultural validity.

Another commonly investigated instrument was the MADRS-S [3, 4, 34–37]. Five of these studies reported Cronbach’s alpha, which is adequate to excellent (.73 to .90, Table 2) [3, 4, 34–36]. Thorndike and colleagues [37] found that the scale consists of 3 factors. Four studies found that the mean score of the MADRS-S does not differ significantly between the online and the paper version [3, 4, 35, 36].

#### Online assessment of GAD

The GAD-7 and two shorter versions were studied among a sample recruited from the general population [38]. The scale showed promising internal consistency, convergent validity and predictive validity. The psychometrics of the GAD-7 were similar among a population of people with hearing loss [6].

#### Online assessment of panic disorder and agoraphobia

Internet interventions for PD/A, such as self-help courses, have been relatively extensively researched. Therefore, Austin and colleagues [39] and Carlbring and colleagues [4] studied the online questionnaires usually employed for such research. They focussed on equivalence of mean scores with paper versions of the same instruments. This equivalence could generally be assumed due to high correlations, but the study of Carlbring [4] found that online versions yield significantly lower mean scores for the Body Sensations Questionnaire (BSQ) and Agoraphobic Cognitions Questionnaire (ACQ) and higher scores for the Mobility Inventory (MI) subscale Alone. Finally, an agoraphobia screening item augmented with images was found to have adequate criterion validity (AUC .73) [7]. All these studies recruited their samples from the general population.

#### Online assessment of social phobia

Two studies [3, 40] independently investigated the equivalence between online and paper versions of the online versions of the Social Interaction Anxiety Scale (SIAS) and Social Phobia Scale (SPS). Both did not find a difference between formats in mean score, but the factor structure did differ between formats [40], indicating that scores cannot be compared across formats. Adequate to good internal consistency of these scales has also been found in three studies [3, 40, 41], and adequate convergent validity of the SIAS in two [40, 41]. Lindner and colleagues revised item 14 of the SIAS, because the original item only applied to heterosexual people. This change did not alter the internal consistency or convergent validity of the scale [41]. The study of Hedman and colleagues [3] recruited people classified with social phobia, but more research among patient groups is recommended.

#### Online assessment of specific phobia

Two of the transdiagnostic screening measures [18, 21] included specific phobia. These showed poor criterion validity for specific phobia. One instrument, the Flight Anxiety Situations Questionnaire (FAS), has been studied for aviophobia [42]. This study showed near perfect criterion validity (AUC .99). Considering aviophobia is only one of many different specific phobias, much more development is needed in this area.

#### Online assessment of OCD

Four instruments for OCD have been studied, all in the US and among the general population [43–45]. Each instrument was studied only once. Williams and colleagues [45] investigated differential item functioning between black and white Americans, finding significant differences for the Padua Inventory (PI). Next to these 4 instruments, the WSQ [18] and the CIDI-SF [21] also screen for OCD.

#### Online assessment of PTSD

Like instruments for OCD, 4 instruments for PTSD have been studied, all in the US and among the general population [31, 46–48]. The transdiagnostic WSQ [18] also screens for PTSD. One additional study investigated an instrument for perinatal PTSD [49]. Miller and colleagues [47] checked the factor structure of their measure for PTSD (National Stressful Events Survey) using item-response theory. The factor structure was confirmed, but the items of the instrument may cover too narrow a range of the latent factors.

#### Online assessment of worry and stress

The PSWQ, assessing worry, was studied twice [20, 50]. These studies found slightly differing values for internal consistency (.73 and .88). We found one study on an instrument that assesses stress [51].

#### Online assessment of suicidal ideation and self-harm

We found one study on an instrument that assesses self-harm. [52] This study used Rasch analysis to further confirm the factors of the Inventory of Statements About Self-injury (ISAS), obtained by factor analysis, and their unidimensionality. Furthermore, we found two single-item measures for suicidal ideation, being item 9 of the BDI-II and item 9 of the MADRS-S [36]. Item 9 of the online BDI-II yielded lower scores than item 9 of the paper version of the BDI-II [36]. The WSQ [18] also contains an item that screens for suicidal ideation, but the validity of this item was not investigated (also see [53]).

### Generalisability and risk of bias

The sample sizes of the included studies were generally adequate for analysing psychometric properties. Nine studies contained over 1000 participants. The other studies in the tables (*n* = 46) had an average sample size of 261 participants. A sample size below 100 was found in 10 studies, which generally gives too little statistical power for psychometric analyses [54]. It should be noted that required sample sizes differ per number of items and type of analysis. Most results could be biased due to selectively missing data. Two studies reported missing data and included numbers. In 33 studies, the amount of missing data was not specifically reported, but could be deduced or estimated. Missing data were not reported by or could not be deduced in 21 studies (see Additional file 3). Overall, COSMIN quality ratings of ‘Excellent’ were rare and ‘Poor’, ‘Fair’ and ‘Good’ ratings were equally common. Instead of adding the COSMIN ratings to the tables and Additional file 3, we decided to report the characteristics the ratings are based on, because the ratings do not always do justice to a study’s quality. The study characteristics give an objective and interpretable indication of the robustness and generalisability of a study’s findings. Lastly, 47 of the 62 instruments were investigated in only one study (Tables 1, 2, 3 and 4), so the robustness of the psychometric properties of these instruments relies heavily on the aspects of the individual studies and cannot be easily generalised to other populations or settings.

## Discussion

This review systematically studied the scientific literature on the psychometrics of online instruments that measure common mental health disorders. We report characteristics of 62 instruments. Most of these instruments were investigated among samples recruited from the general population. We found at least one online measure for each of the included mental health disorders and symptoms. The results are scattered over different instruments and different characteristics and, therefore, can be synthesised for only a few instruments. We found few instruments that measure specific phobia, stress, worry self-harm and suicidal ideation. There were no studies that reported that the questionnaires were completed on cellular phones or smartphones.

The CES-D is the most well-studied online instrument and there is evidence for adequate psychometric properties among samples recruited from the general population. The MADRS-S has been well-studied as well, mostly showing mean score equivalence between online and paper versions. Finally, the HADS is the only instrument that was investigated among both the general population and two patient populations, showing adequate psychometric properties.

Ideally, two or more online instruments would be available for each disorder, with all of their characteristics examined in several studies, among various populations. There are clear gaps in the tables presented in this study, which warrant further research and development. The psychometric properties measurement error and responsiveness were not reported for any instrument. Furthermore, while there is an abundance of online instruments for depressive symptoms, there is a shortage of instruments for other disorders. Although a few new instruments have been developed in the meantime, e.g. for suicidal ideation [55], more instruments are needed.

Equivalence between paper and online versions of an instrument has mostly been studied in the form of equivalence of mean scores by correlations and t-tests. We can conclude that correlations are high and differences are small. However, mean score equivalence is only one aspect of measurement invariance. Two studies conducted a broad range of measurement invariance tests [33, 40]. While Yu and Yu [33] found only a negligible difference in the mean score of the somatic factor of the CES-D, Hirai and colleagues [40] found that factor structures of the SIDAS and SPS differ between formats. Differing factor structures indicate that different constructs are assessed and scores cannot be compared across formats. It is important to note that possibly not only the format differs between paper and online versions, but the setting as well. Online questionnaires can be completed at the participant’s home on a device (s) he is familiar with. In the study of Yu and Yu [33], participants completed the paper questionnaires at home, while in the study of Hirai and colleagues [40], participants completed the paper questionnaires in a lab. It is recommended to study inter-format equivalence in one setting, and to use a broad range of measurement invariance aspects, e.g. using multiple-group confirmatory factor analysis [56].

This systematic review has some limitations. Firstly, we may not have included all studies on psychometrics of online instruments for common mental health disorders, because there may be studies that applied online assessment without mentioning it in the title or abstract. Online assessment is increasingly common and increasingly less important to mention. Secondly, we decided not to label the quality of the included studies, even though a quality assessment is common practice in systematic reviews. Because psychometric properties are dependent on study characteristics, it is more insightful to inspect these characteristics in order to decide whether an instrument has been investigated well enough for the purpose, population and setting one wants to use it for. Thirdly, our search has been updated up to January 1^st^ 2014 and several psychometric studies on online instruments have been published since. Finally, our search strings (Additional file 2) can be made more comprehensive by adding ‘distress’, ‘mhealth’ and ‘response processes’. The omission of these terms have not impacted our results, however.

Future psychometric studies are encouraged to investigate and explore different devices, formats and media. Only one study in our review [37] investigated the effects of different formats of online questionnaires and the preferences of the participants. An instrument’s format, e.g. the layout, design, font type and the number of items per page, interacts with its content and with the characteristics of the individual who completes the items. [57] Different formats could also include other media than text, such as audio, images and video, see e.g. [6] and [7]. Another area to explore is measurement by smartphones, which we did not encounter in the included studies. The validity of measurement by smartphone applications has been studied in other fields, such as psychotic symptoms. [58] An advantage of measurement by smartphones is that it enables momentary assessment, opposed to retrospective assessment, because an individual can have access to his/her smartphone all day long.

## Conclusions

We found at least one online measure for each of the included mental health disorders and symptoms, and there is mounting evidence for adequate psychometric properties of common instruments such as the CES-D, MADRS-S and HADS. Overall, the results are scattered over different instruments and different characteristics, and much work still has to be done in this field. With this systematic review we provide a framework for future research into psychometrics of online instruments. Furthermore, our overview of instruments can guide professionals when choosing an instrument for assessing common mental health disorders online. The tables (Additional file 3) provided with this systematic review are free to use and expand. We encourage researchers to fill in the missing data and to add innovative instruments.

## Additional files

Additional file 1:
**PRISMA Checklist.** (DOC 60 kb) Additional file 2:
**Search strings.** (DOCX 18 kb) Additional file 3:
**Included studies and extracted data.** (XLS 117 kb)
