# Supplementary material for: Validation of online psychometric instruments for common mental health disorders: a systematic review
Source: BMC Psychiatry. 2016 Feb 25;16:45. doi: 10.1186/s12888-016-0735-7 (PMC4766750; doi:10.1186/s12888-016-0735-7)
Supplement: Additional file 2: — Search strings. (DOCX 18 kb) [file 12888_2016_735_MOESM2_ESM.docx]

**PubMed search**

((("Anxiety"[Mesh] OR "Anxiety Disorders"[Mesh] OR anxiet* [tiab] OR anxious* [tiab] OR phobi* [tiab] OR panic* [tiab] OR "obsessive-compulsive" [tiab] OR "post-traumatic stress"[tiab] OR "posttraumatic stress" [tiab] OR agoraphobi* [tiab] OR "Depression"[Mesh] OR "Depressive Disorder"[Mesh] OR depression* [tiab] OR depressiv* [tiab] OR "Self-Injurious Behavior" [Mesh] OR "self-injurious behavior"[tiab] OR "self-injurious behaviour"[tiab] OR self-harm [tiab] OR selfharm [tiab] OR automutilat* [tiab] OR auto-mutilation [tiab] OR "suicide" [Mesh] OR suicid* [tiab] OR "Stress, psychological" [Mesh] OR "psychological stress" [tiab])) AND ("Internet"[Mesh] OR internet [tiab] OR online [tiab] OR web [tiab] OR internet-based [tiab] OR internetbased [tiab] OR online-based [tiab] OR internet-delivered [tiab] OR web-based [tiab] OR webbased OR “cellular phone” [Mesh] OR smartphone* [tiab] OR “smart phone” [tiab] OR “mobile phone” [tiab] OR “cellular phone” [tiab] OR e-health [tiab] OR eHealth [tiab] OR “e-mental health” [tiab] OR “Telemedicine” [Mesh] OR telemedicine [tiab] OR telehealth [tiab] OR “mobile health” [tiab])) AND (((instrumentation[sh] OR methods[sh] OR "Validation Studies"[pt] OR "Comparative Study"[pt] OR "psychometrics"[MeSH] OR psychometr*[tiab] OR clinimetr*[tw] OR clinometr*[tw] OR "outcome assessment (health care)"[MeSH] OR "outcome assessment"[tiab] OR "outcome measure*"[tw] OR "observer variation"[MeSH] OR "observer variation"[tiab] OR "Health Status Indicators"[Mesh] OR "reproducibility of results"[MeSH] OR reproducib*[tiab] OR "discriminant analysis"[MeSH] OR reliab*[tiab] OR unreliab*[tiab] OR valid*[tiab] OR "coefficient of variation"[tiab] OR coefficient[tiab] OR homogeneity[tiab] OR homogeneous[tiab] OR "internal consistency"[tiab] OR (cronbach*[tiab] AND (alpha[tiab] OR alphas[tiab])) OR (item[tiab] AND (correlation*[tiab] OR selection*[tiab] OR reduction*[tiab])) OR agreement[tw] OR precision[tw] OR imprecision[tw] OR "precise values"[tw] OR test-retest[tiab] OR (test[tiab] AND retest[tiab]) OR (reliab*[tiab] AND (test[tiab] OR retest[tiab])) OR stability[tiab] OR interrater[tiab] OR inter-rater[tiab] OR intrarater[tiab] OR intra-rater[tiab] OR intertester[tiab] OR inter-tester[tiab] OR intratester[tiab] OR intra-tester[tiab] OR interobserver[tiab] OR inter-observer[tiab] OR intraobserver[tiab] OR intra-observer[tiab] OR intertechnician[tiab] OR inter-technician[tiab] OR intratechnician[tiab] OR intra-technician[tiab] OR interexaminer[tiab] OR inter-examiner[tiab] OR intraexaminer[tiab] OR intra-examiner[tiab] OR interassay[tiab] OR inter-assay[tiab] OR intraassay[tiab] OR intra-assay[tiab] OR interindividual[tiab] OR inter-individual[tiab] OR intraindividual[tiab] OR intra-individual[tiab] OR interparticipant[tiab] OR inter-participant[tiab] OR intraparticipant[tiab] OR intra-participant[tiab] OR kappa[tiab] OR kappa's[tiab] OR kappas[tiab] OR repeatab*[tw] OR ((replicab*[tw] OR repeated[tw]) AND (measure[tw] OR measures[tw] OR findings[tw] OR result[tw] OR results[tw] OR test[tw] OR tests[tw])) OR generaliza*[tiab] OR generalisa*[tiab] OR concordance[tiab] OR (intraclass[tiab] AND correlation*[tiab]) OR discriminative[tiab] OR "known group"[tiab] OR "factor analysis"[tiab] OR "factor analyses"[tiab] OR "factor structure"[tiab] OR "factor structures"[tiab] OR dimension*[tiab] OR subscale*[tiab] OR (multitrait[tiab] AND scaling[tiab] AND (analysis[tiab] OR analyses[tiab])) OR "item discriminant"[tiab] OR "interscale correlation*"[tiab] OR error[tiab] OR errors[tiab] OR "individual variability"[tiab] OR "interval variability"[tiab] OR "rate variability"[tiab] OR (variability[tiab] AND (analysis[tiab] OR values[tiab])) OR (uncertainty[tiab] AND (measurement[tiab] OR measuring[tiab])) OR "standard error of measurement"[tiab] OR sensitiv*[tiab] OR responsive*[tiab] OR (limit[tiab] AND detection[tiab]) OR "minimal detectable concentration"[tiab] OR interpretab*[tiab] OR ((minimal[tiab] OR minimally[tiab] OR clinical[tiab] OR clinically[tiab]) AND (important[tiab] OR significant[tiab] OR detectable[tiab]) AND (change[tiab] OR difference[tiab])) OR (small*[tiab] AND (real[tiab] OR detectable[tiab]) AND (change[tiab] OR difference[tiab])) OR "meaningful change"[tiab] OR "ceiling effect"[tiab] OR "floor effect"[tiab] OR "item response model"[tiab] OR irt[tiab] OR rasch[tiab] OR "differential item functioning"[tiab] OR dif[tiab] OR "computer adaptive testing"[tiab] OR "item bank"[tiab] OR "cross-cultural equivalence"[tiab])))

**PsycINFO search**

( DE “anxiety disorders” OR DE Anxiety OR TI Anxiet* OR AB Anxiet* OR TI anxious* OR AB Anxious* OR TI panic* OR AB panic* OR DE Phobias OR TI phobi* OR AB phobi* OR DE “posttraumatic stress disorder” OR TI “post-traumatic stress” OR AB “post-traumatic stress” OR TI “posttraumatic stress” OR AB “posttraumatic stress” OR TI agoraphobi* OR AB agoraphobi* OR DE "Depression (Emotion)" OR TI depression* OR AB depression* OR DE "Major Depression" OR TI depressiv* OR AB depressiv* OR DE "Obsessive Compulsive Disorder" OR TI “obsessive compulsive” OR AB “obsessive compulsive” OR DE "Suicide" OR TI suicid* OR AB suicid* OR DE "Self Injurious Behavior" OR DE "Self Destructive Behavior" OR DE "Self Mutilation" OR TI “self-injurious behavior “ OR AB “self-injurious behavior” OR TI “self-injurious behaviour” OR AB “self-injurious behaviour “ OR TI self-harm OR AB self-harm OR TI selfharm OR AB selfharm OR TI automutilat* OR AB automutilat* OR TI auto-mutilat* OR AB auto-mutilat* OR DE "Psychological Stress" OR TI “psychological stress” OR AB “psychological stress” ) AND ( DE "Internet" OR TI internet OR AB internet OR DE "Online Therapy" OR TI online OR AB online OR TI internet-based OR AB internet-based OR TI internetbased OR AB internetbased OR TI online-based OR AB online-based OR TI web OR AB web OR TI webbased OR AB webbased OR TI web-based OR AB web-based OR TI internet-delivered OR AB internet-delivered OR DE "Telemedicine" OR TI telemedicine OR AB telemedicine OR TI telehealth OR AB telehealth OR TI teletherapy OR AB teletherapy OR TI e-therapy OR AB e-therapy OR TI e-health OR AB e-health OR TI "e-mental health" OR AB "e-mental health" OR DE "Cellular Phones" OR TI cellular phone* OR AB cellular phone* ORTI cellular phone* OR AB cellular phone* OR TI cell phone* OR AB cell phone* OR TI smartphone* OR AB smartphone* OR TI mobile phone* OR AB mobile phone* OR TI smart phone* OR AB smart phone* OR TI cybercounseling OR AB cybercounseling OR TI eHealth OR AB eHealth OR TI mobile health OR AB mobile health ) AND ( ( CC 2200 OR CC 2220 OR CC 2222 OR CC 2224 OR CC 2225 OR CC 2226 OR CC 2227 OR CC 2228 OR CC 2260 ) OR ( TI psychometric* OR AB psychometric* OR TI clinimetr* OR AB clinimetr* OR TI clinometr* OR AB clinometr* OR TI validat* OR AB validat* OR TI validity OR AB validity OR TI reliability OR AB reliability OR TI reliabl* OR TI measur* OR TI screen* OR TI "internal consistency" OR AB "internal consistency" OR AB cronbach OR TI test-retest OR AB test-retest OR (AB test AND AB retest) OR TI sensitiv* OR AB sensitiv* OR TI specificity OR AB specificity OR TI "predictive value" OR AB "predictive value" OR TI responsiveness OR AB responsiveness OR TI "questionnaire" OR TI "structural equation modeling" OR AB "structural equation modeling" OR TI "structural equation modelling" OR AB "structural equation modelling" OR TI "factor analysis" OR AB "factor analysis" OR TI "factor structure" OR AB "factor structure" OR TI factorial OR AB factorial OR TI "principal component" OR AB "principal component" OR (AB item AND (AB correlation* OR AB selection* OR AB reduction)) OR TI "differential item functioning" OR AB "differential item functioning" OR TI "item response" OR AB "item response" OR TI Rasch OR AB Rasch OR TI "cross-cultural equivalence" OR AB "cross-cultural equivalence" OR DE "Adaptive Testing" OR DE "Behavioral Assessment" OR DE "Biographical Inventories" OR DE "California Psychological Inventory" OR DE "Central Tendency Measures" OR DE "Classical Test Theory" OR DE "Clinical Judgment (Not Diagnosis)" OR DE "Cloze Testing" OR DE "Cognitive Assessment" OR DE "Computer Assisted Diagnosis" OR DE "Computer Assisted Testing" OR DE "Conjoint Measurement" OR DE "Consistency (Measurement)" OR DE "Content Analysis (Test)" OR DE "Cutting Scores" OR DE "Diagnosis" OR DE "Diagnostic and Statistical Manual" OR DE "Diagnostic Interview Schedule" OR DE "Differential Diagnosis" OR DE "Difficulty Level (Test)" OR DE "Educational Diagnosis" OR DE "Educational Measurement" OR DE "Essay Testing" OR DE "Factor Structure" OR DE "Forced Choice (Testing Method)" OR DE "Forensic Evaluation" OR DE "Frequency Distribution" OR DE "General Health Questionnaire" OR DE "Health Screening" OR DE "Homogeneity of Variance" OR DE "International Classification of Diseases" OR DE "Interrater Reliability" OR DE "Inventories" OR DE "Item Analysis (Test)" OR DE "Item Content (Test)" OR DE "Item Response Theory" OR DE "Likert Scales" OR DE "Mail Surveys" OR DE "Medical Diagnosis" OR DE "Multiple Choice (Testing Method)" OR DE "Neuropsychological Assessment" OR DE "Predictability (Measurement)" OR DE "Prognosis" OR DE "Psychiatric Evaluation" OR DE "Psychodiagnosis" OR DE "Psychodiagnostic Interview" OR DE "Psychodiagnostic Typologies" OR DE "Psychometrics" OR DE "Psychological Assessment" OR DE "Psychological Screening Inventory" OR DE "Questionnaires" OR DE "Rating Scales" OR DE "Rating" OR DE "Repeated Measures" OR DE "Research Diagnostic Criteria" OR DE "Retention Measures" OR DE "Scaling (Testing)" OR DE "Scoring (Testing)" OR DE "Screening Tests" OR DE "Screening" OR DE "Selection Tests" OR DE "Sentence Completion Tests" OR DE "Standard Scores" OR DE "Statistical Measurement" OR DE "Statistical Norms" OR DE "Statistical Probability" OR DE "Statistical Reliability" OR DE "Statistical Validity" OR DE "Structured Clinical Interview" OR DE "Surveys" OR DE "Telephone Surveys" OR DE "Test Administration" OR DE "Test Bias" OR DE "Test Construction" OR DE "Test Forms" OR DE "Test Interpretation" OR DE "Test Items" OR DE "Test Norms" OR DE "Test Reliability" OR DE "Test Scores" OR DE "Test Standardization" OR DE "Test Validity" OR DE "Testing Methods" OR DE "Testing" OR DE "Variability Measurement" ) )
